# Atlanta Academy of Medicine

**Published:** 1873-07

**Authors:** Jas. P. Baird

**Affiliations:** Reporter


### ATLANTA ACADEMY OF MEDICINE.

JAS. B. BAIRD, M.D., REPORTER.

*Extract from the Minutes, March* 17, 1873—*Dr. Jno. G. Westmoreland,* 1*st Vice-President, in the Chair.*

Dr. W. F. Westmoreland, the essayist of the evening, opened the discussion of the question as previously announced—viz, syphilis and chancroid—by calling attention to the following points:

1. The relation they bear to each other.
2. Differential diagnosis, or distinctive characteristics of each.
3. Treatment of each.
4. If impossible to determine the character of the local development, the most rational plan of treatment to be pursued.

Chancre, or syphilis, has only been understood by the profession since the fifteenth century. Up to that time the constitutional disease, syphilis, was never heard of, though chancroid, or soft chancre, was recognized and described long before that time, and treated better than by many surgeons of the nineteenth century.

He would not enter into the discussion of the origin of syphilis. Many think that it is indigenous to America, but Chinese medical literature furnishes evidence of the existence of syphilis in that country centuries before Christ. He, however, desired to impress the fact that a distinction between the two diseases was made. Up to twenty years ago, the opinion of surgeons was that the same poison produced different effects in different persons. As late as 1854 it was taught, by the great Ricord, that the virus producing these lesions was one and the same, modified, however, in its results by individual peculiarities of constitution. In 1854 or 1855 M. Clerc admitted with Ricord the unity of the virus producing the two sores, but denied that it was modified by constitutional peculiarities. He held that soft chancre, or chancroid, bore the same relation to the true chancre, or syphilis, that varioloid does to variola. He maintained that if a subject who had syphilis, or had had it, should be inoculated with syphilitic virus, that a chancroid would be produced; and further, that if a non-infected person should be inoculated with virus from a chancroid produced in the manner above described, that a similar sore would result.

A few years later Bassereau declared the virus that produced hard and soft chancre was as distinct as the virus producing chancre and gonorrhœa; that virus from a chancroid produced a chancroid, and never the constitutional disease; and that the virus from a chancre produced a like sore, or syphilis. An important point in the differential diagnosis is the stage of incubation. The chancroid has none. As soon as inoculation takes place, the sore is produced in three or four days. Chancre or syphilis has a period of incubation ranging from twenty-five to one hundred and twenty-five days. Rarely less than twenty-five days.

*Character of the Local Development.*—In chancroid the ulcers are generally multiple. If at first only one point is inoculated, other sores are soon produced by the secretions from the original sore. In the majority of cases of chancre but one point is inoculated, and chancres are not auto-inoculable.

*Anatomical Character.*—In chancroids a loss of tissue is a prominent feature, from the first development, with a tendency to phagedæna. In chancre there is usually but a very trifling loss of tissue, but an increase of substance, and consequently induration. Chancre, unless from accidental causes, or constitutional defects, does not tend to phagedæna.

*Secretion.*—In chancroid profuse—not only serous, but also pus from broken down tissue. In chancre the secretion is scanty or wanting. Statistics prove that chancroids are more contagious than chancres. Thus, in one thousand cases, they stand in the proportion of eight hundred and forty-five chancroids to one hundred and fifty-five chancres.

*Greatly increased sensibility* is another peculiarity of the chancroid, while the chancre may be absolutely painless, and is frequently, on this account, not recognized by the patient.

*Induration.*—In chancroid there is little or no induration. In chancre it is conspicuous. Hence the terms respectively applied—soft and hard, or indurated and non-indurated chancre.

*Affection of Glands.*—When the inguinal glands, in chancroid, are involved, it is through sympathy, or transmission of the virus to them by the lymphatics. In the majority of cases but one gland is involved, but it is acutely inflamed, and, if not properly treated, a suppurating bubo results. In syphilis, generally, the chain of glands in one or both groins become enlarged before the disease is suspected. The glands do not inflame acutely, as in chancroid, unless as the result of irritating treatment applied to the chancre, or phagedenic ulceration of the primary sore.

*Prognosis.*—Chancroid is merely a local disease; syphilis is constitutional.

*Treatment.*—Chancroid requires only local treatment, and if left to itself there are no constitutional symptoms, and recovery takes place without any consecutive lesion. If constitutional treatment be adopted, it is for complications. It is wrong to use mercury. It does no good, but does harm. Use caustics, topically, of sufficient strength to destroy the virus. Nitric acid is the best. Be cautious and thorough in its application. See that the whole surface of the sore sloughs.

In chancre, or syphilis, we have a different condition. The constitution is infected, as the poison has been in the system from twenty-five to one hundred and twenty-five days before the first symptom presents itself. Mercury is the only rational plan of treatment. Not only does it eradicate, but it prevents the development of the disease. Will not stop to compare different plans of treatment, but mercury in the early, the primary stages; and later, in the secondary and tertiary stages, iodide of potassium, not in ten, fifteen, or twenty grain doses, but fifty, or even one hundred grains daily.

As to the fourth point, Ricord adopted the most rational plan of treatment. If the nature of the disease is not clear do not treat at all, but do nothing—remembering that the day can not be far off when the disease will be sufficiently developed to show what it is. Do not blindly fill the patient with mercury, for it may produce lesions as serious as syphilis itself. A few days, or weeks, will indicate the proper course to pursue.

